# Displaced Pediatric Supracondylar Fracture (Gartland Type II and III): The Impact of Wire Fixation Type

**DOI:** 10.7759/cureus.72110

**Published:** 2024-10-22

**Authors:** Sanjay Jain, Deeraj Loganathan, Zain Habib, Rama Mohan

**Affiliations:** 1 Trauma and Orthopedics, North Manchester General Hospital, Manchester, GBR; 2 Trauma and Orthopedics, Manchester University NHS Foundation Trust, Manchester, GBR

**Keywords:** complications, k-wire fixation, pediatric type iii supracondylar fractures, radiographic outcomes, supracondylar humeral fractures

## Abstract

Introduction

Supracondylar fractures are common in children. Percutaneous K-wire fixation is an accepted standard treatment for displaced supracondylar fractures in children, but the ideal wire configuration remains controversial. This study aimed to review the radiographic outcome and complications of Gartland type II and type III supracondylar fractures treated by the crossed and lateral K-wire fixation.

Methods

Seventy-five cases were retrospectively reviewed, 41 and 34 in crossed and lateral K-wire fixation groups, respectively. We studied patient demographics, fracture characteristics, different operative variables, and complications between the two groups.

Results

No significant difference was noted in patient demographics and the size of the wire used between the two groups. More type III fractures were fixed with crossed wires, 31 vs. 15 (75% vs. 44%, p = 0.019). Higher open reduction was also noted in the crossed-wire group. Loss of reduction between the crossed and the lateral groups was not significantly different, 15 vs. 13 (36.6% vs. 38.2%). The crossed-wire group had more iatrogenic nerve injury than the lateral wire group, 9 vs. 1 (22% vs. 3%, p = 0.0185). Higher complications were observed in the crossed-wire group than in the lateral-wire group, mainly due to iatrogenic nerve injury, 19 vs. 5 (46.3% vs. 14.7%, p = 0.0052). Higher technical errors of wire fixation were noted with lateral wire fixation than with crossed-wire fixation, 28 vs. 12 (82% vs. 29%, p = 0.00001).

Conclusions

Higher complications were noted with crossed wires, primarily due to iatrogenic nerve injury. Between the crossed and lateral wire groups, there were no statistically significant differences in loss of reduction and other complications. We support lateral wiring with appropriate techniques in treating types II and III supracondylar fractures to avoid iatrogenic nerve injury and other complications.

## Introduction

Supracondylar humeral fractures are the most common fractures affecting the elbow in children [1]. The incidence is highest in the five- to eight-year age range [2]. The mechanism is usually caused by a fall onto an outstretched hand with a full extension of the elbow, noted in 97-99% of cases [2]. Supracondylar fractures are usually classified in accordance with the Gartland system of classification into three types: type I being non-displaced, type II being displaced but with an intact posterior cortex, and type III being displaced and without any cortical contact. Type II fracture was further subclassified into type IIa with posterior angulation only and type IIb posteriorly angulated and rotated [3]. Closed reduction and percutaneous K-wire fixation are widely accepted treatments for displaced humeral supracondylar fractures in children, but the ideal configuration of the wires continues to be debated.

Our study aimed to assess and review the radiographic outcome and complications of displaced (Gartland type II and type III) supracondylar fractures managed by either crossed or lateral K-wire fixation.

## Materials and methods

**Figure 1:**
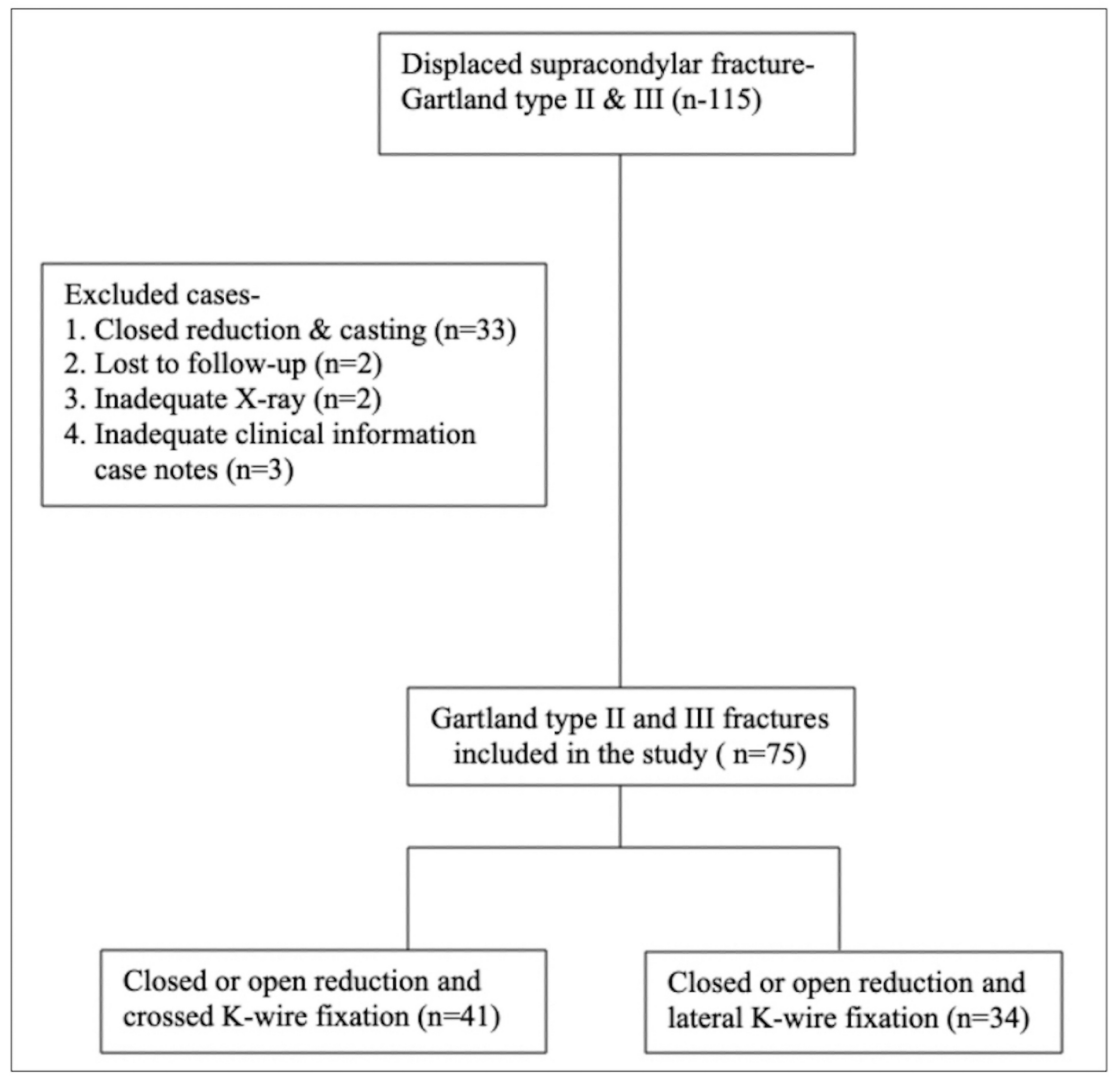
Flowchart of patient inclusion

About 115 children with displaced supracondylar (Gartland type II and III) fractures were admitted for surgery in our institution between January 2014 and July 2020. Finally, 75 cases of displaced supracondylar fractures treated with K-wire, were included in the study after our selection criteria as depicted in Figure 1. Patients were excluded if they were lost to follow-up, due to inadequate X-rays or clinical information, or if patients had been treated conservatively with closed reduction and casting.

The operating surgeon decided on the wire size and wire configuration. The back slab was applied after K-wire fixation. The children were discharged from the hospital, usually on the same day or the next day, and were seen in the clinic 7-10 days after the operation for a pin site check, complete cast, and X-rays. The child was reviewed again after 2-3 weeks for repeat X-rays; removal of cast, wires, and motion was encouraged. A physiotherapy referral was done if elbow stiffness was developing. The average follow-up in our study was 13.7 ± 8.5 weeks.

Electronic medical records and radiographs were reviewed to determine patient demographics, injury type, types of fracture, associated other fractures, preoperative neurovascular involvement, the timing of surgery, reduction method (closed or open), K-wire configuration, number and size of K-wires, loss of reduction, faulty wiring technique, iatrogenic nerve injuries, infection, and any other complications.

**Figure 2:**
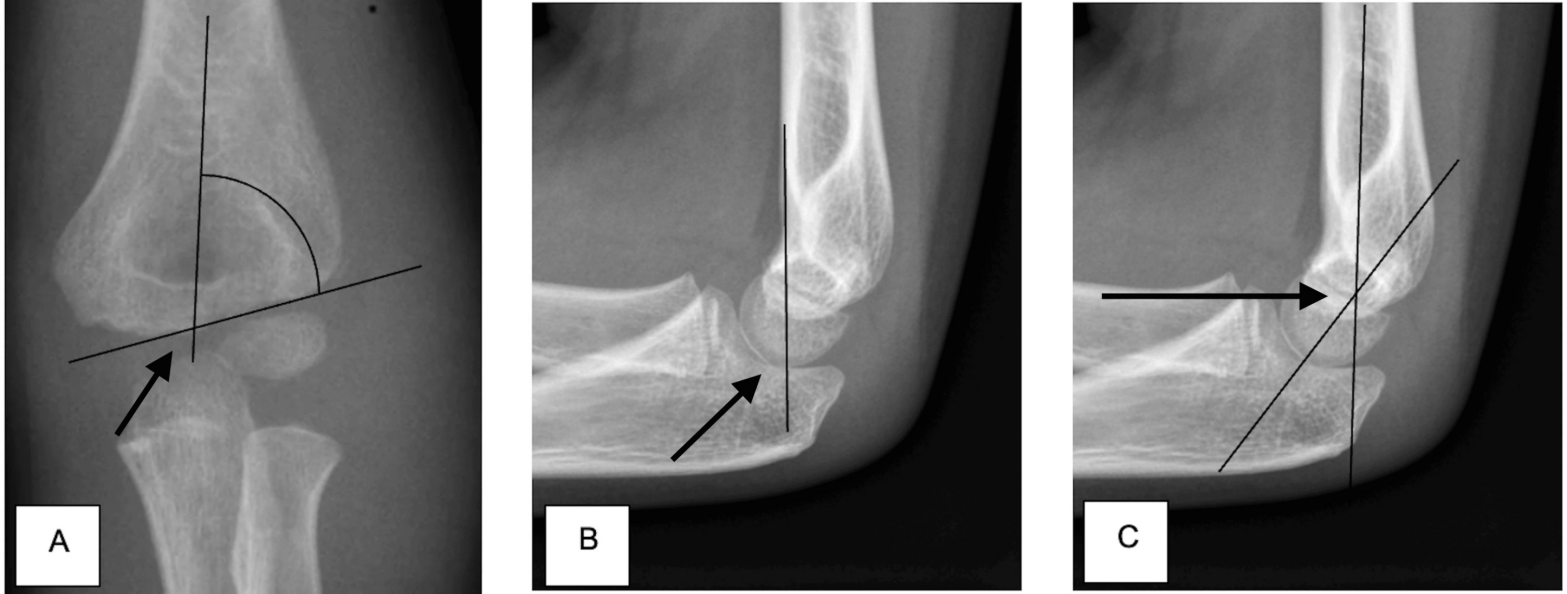
Parameters for judging loss of fracture reduction (A) Bauman’s angle (BA) - this angle is formed by the humeral axis and capitellar physis on the AP radiograph. (B) Anterior humeral line (AHL) - a line drawn along the anterior border of the distal humerus in the proximal fragment on the lateral radiograph; the line usually intersects the capitellum. (C) Humerocapitellar angle (HCA) - the angular relationship between the humeral shaft and capitellum on the lateral radiograph.

Loss of fracture reduction was determined by comparing the perioperative and postoperative follow-up X-rays. We reviewed loss of reduction by evaluating changes in the Bauman angle (BA) >12 degrees in the coronal plane and the humeral-capitellar angle (HCA) >10 degrees in the sagittal plane. We also assessed if the anterior humeral line (AHL) was not intersecting the capitellum in the sagittal plane. Combining these three parameters we were able to judge loss of fracture reduction comprehensively which is demonstrated below in Figure 2 [4,5].

**Figure 3:**
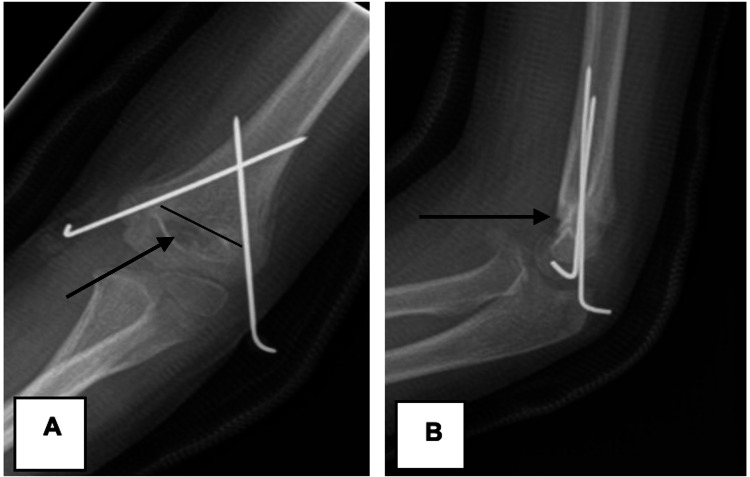
Radiographic example of adequate fixation using the crossed K-wire technique (A) Anteroposterior and (B) lateral radiograph showing adequate reduction and fixation of the displaced supracondylar fracture with crossed wires (bi-cortical fixation with adequate separation at the fracture site).

Bi-cortical fixation and separation of wires at the fracture site were considered adequate crossed K-wire fixation techniques, as demonstrated below in Figure 3.

**Figure 4:**
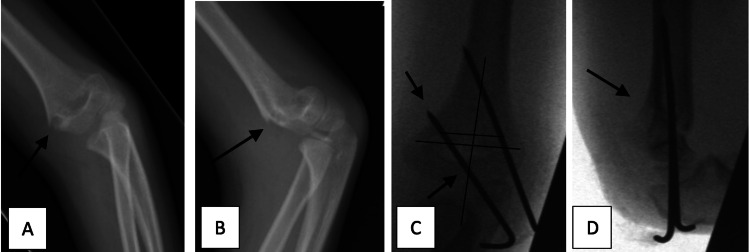
Radiographic example of adequate fixation using lateral K-wires technique (A, B) Preoperative AP and lateral radiographs with displaced supracondylar fracture. (C, D) Intra-operative anteroposterior and lateral radiograph showing bi-cortical, bi-columnar, divergent lateral wire fixation and separation of wires >1/3 circumference of the humerus at the fracture site.

The lateral wiring technique was considered to be correct if wires were bi-columnar, bi-cortical, and divergent, with separation of wires more than a 1/3 of the circumference of the humerus, at the fracture site (4 and 5). This is demonstrated below in Figure 4.

We regarded it as a technical error of wire fixation if these criteria were not fulfilled.

Statistical analysis was performed using open-source online resources. Data of all variables were analyzed to determine differences between the crossed and lateral K-wire fixation groups. Statistical significance was tested using Fisher's exact test, and a p-value less than 0.05 was set as the threshold for statistical significance.

## Results

Out of 75 children, 34 (45.3%) had lateral, and 41 (54.7%) had crossed-wire fixation. The mean age of children was 6.4 ± 2.2 years in the crossed-wire group and 6.3 ± 2.1 years in the lateral-wire group. Of 44 children in the crossed-wire group, 25 (56%) were boys, and the right arm was involved in 26 (58.5%) of cases. Of 34 children in the lateral wire group, 25 (73.5%) were boys, and the right arm was involved in 15 (44.1%) of cases. There were 31 (75%) Gartland type III, three (7.3%) were type IIa, and seven (17%) were type IIb fractures in the crossed-wire group, and 15 (44.1%) were Gartland type III, seven (20.6%) were type IIa, and 12 (35.3%) were type IIb fractures in the lateral wire group. In our study, a significantly higher percentage of type III fractures were treated with crossed K-wire fixation than lateral wire fixation, 31 vs. 15 (75% vs. 44%, p = 0.019). Most children in our study were operated on in our series within 24 hours of admission. These demographics are presented below in Table 1.

**Table 1:** Demographics and variables with crossed and lateral wire fixation of supracondylar fracture A p-value is considered significant (p < 0.05); significant p-values are denoted with *. Age presented as mean ± SD, sex presented as N (%), side presented as N (%), fracture type (IIa, IIb, and III) presented as N (%), type of reduction presented as N (%), open fracture presented as N (%), preoperative nerve injury presented as N (%), associated fracture presented as N (%) pre-op: preoperative

Variable	Crossed wire (n = 41) N (%) / mean ± SD	Lateral wire (n = 34) N (%) / mean ± SD	p-value
Age	6.4 ± 2.2 years	6.3 ± 2.1 years	0.842
Sex: Male	23 (56%)	25 (73.5%)	0.1205
Sex: Female	18 (43.9%)	9 (26.5%)	0.1205
Side: Right	24 (58.5%)	15 (44.1%)	0.215
Side: Left	17 (41.5%)	19 (55.8%)	0.215
Fracture type IIa	3 (7.3%)	7 (20.6%)	0.019*
Fracture type IIb	7 (17%)	12 (35.3%)	0.019*
Fracture type III	31 (75%)	15 (44.1%)	0.019*
Time to surgery: <24 hours	39 (95%)	29 (85%)	-
Time to surgery: >24 hours	2 (4.8%)	5 (14.7%)	-
Type of reduction: Open	14 (34.1%)	3 (8.8%)	0.0148*
Type of reduction: Closed	27 (65.9%)	31 (91.2%)	0.0148*
Open fracture	1 (2.4%)	0	-
Pre-op nerve injury: Median nerve (MN)	1 (2.4%) (sensory)	0	-
Pre-op nerve injury: Anterior interosseous nerve (AIN)	1 (2.4%)	0	-
Pre-op nerve injury: Ulnar nerve (UN)	0	0	-
Pre-op nerve injury: Combination and types	1 UN + MN (2.4%) (sensory)	0	-
Associated fracture: Distal radius fracture	1 (2.4%)	1 (2.9%)	-

In our study, 17 (22.7%) children needed an open fracture reduction. We noted a higher open reduction in the crossed wire than lateral wire fixation, 14 vs. 3 (34% vs. 8.8%, p = 0.0148), respectively. Two (2.6%) cases, one in each group, had associated distal radius fractures. One child (2.4%) had an open fracture in the crossed-wire group. Preoperative nerve palsy was noted in three (7.3%) in the crossed-wire group only, and all recovered fully by 12 weeks. This data is also depicted below in Table 1.

Preoperatively, two patients (2.7%) had a feeble pulse and poor capillary refill, and in both cases, pulse and capillary refill returned to normal after the reduction of fractures. No one had compartment syndrome or required vascular surgery input.

The groups were quite similar in size to the K-wire (1.6/2 mm) used to fix the fractures, and the majority of them, 55 (73.3%) were fixed with two wires in both groups. The distribution of various wire configurations used to treat the fractures in both groups is also shown in Table 2.

**Table 2:** Variables with crossed and lateral wire fixation of supracondylar fracture A p-value is considered significant (p < 0.05); significant p-values are denoted with *. Size of K-wire presented as N (%), loss of reduction presented as N (%), technical errors of wire fixation presented as N (%), mean change of BA and HCA presented as mean ± SD, physiotherapy referral presented as N (%), average follow-up presented as mean ± SD BA: Bauman’s angle; HCA: humerocapitellar angle

Variable	Crossed wire (n = 41) N (%) / mean ± SD	Lateral wire (n = 34) N (%) / mean ± SD	p-value
Size of K-wire: 1.6 mm	20 (48.8%)	15 (44.1%)	0.687
Size of K-wire: 2 mm	21 (51.2%)	19 (55.9%)	0.687
Loss of reduction	15 (36.6%)	13 (38.2%)	0.926
Technical errors in wire fixation	12 (29.3%)	28 (82.4%)	0.00001*
Mean change BA	5.54 ± 4.6	3.5±3.2	0.0322*
Mean change HCA	7.33 ± 7.2	7.25 ± 5.1	0.9567
Physiotherapy referral	16 (39%)	15 (44.1%)	0.814
Average follow up ± SD	13.7 ± 8.5	13.7 ± 8.5	-

Physiotherapy referral was done in 16 cases (39%) and 15 cases (44.1%) in crossed and lateral wire groups, respectively (p = 0.814).

There was no significant difference noted in radiographic loss of reduction between crossed (15, 36.6%) and lateral-wired (13, 38.2%) fixation groups. There was a statistically significant difference (p < 0.00001) noted in the technical error of wire fixation between crossed (12, 29%) and lateral (28, 82%) wire fixation groups, as shown in Table 2.

**Figure 5:**
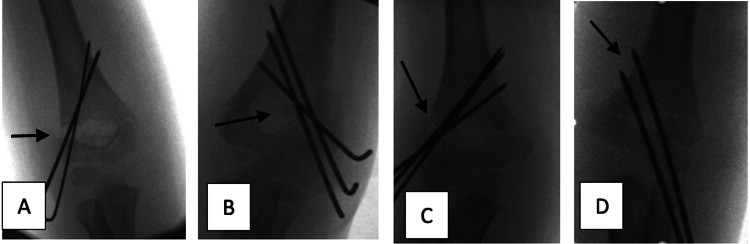
Technical errors of lateral wire fixation (A, B, C, D) Anteroposterior radiograph showing inadequate fixation (using two or three wires) of displaced supracondylar fracture (not bi-columnar, not divergent, and separation of wires <1/3 circumference of the humerus at the fracture site).

Wires not being separated at the fracture site of more than 1/3 of the humerus width, 24 (85.7%) were the most common type of error in the lateral wire group, as depicted by examples in Figure 5.

The proportion of other types of errors for lateral wire fixation is summarized below in Table 3.

**Table 3:** Types of technical errors with the lateral wire fixation technique Types of error variables are presented as N (%).

Types of error	Technical errors of lateral wire fixation (n = 28) N (%)
Not bi-columnar	22 (78.6%)
Not bi-cortical	2 (7.1%)
Not divergent	16 (57.1%)
Wires not separated 1/3 of humerus width at the fracture site	24 (85.7%)

Wires not being bi-cortical were the most common, with nine (75%) types of error in the crossed-wire group. The other types of errors for this patient group are summarized below in Table 4.

**Table 4:** Types of technical errors with crossed-wire fixation technique Types of error variables presented as N (%)

Types of error	Technical errors of crossed-wire fixation (n = 12) N (%)
Not bi-cortical	9 (75%)
Wires not separated at the fracture site	3 (25%)

The mean changes of BA were 5.54 ± 4.6 degrees in the crossed-wire group versus 3.5 ± 3.2 degrees in the lateral-wire group (p = 0.0322). The mean changes of HCA were 7.33 ± 7.2 degrees in the crossed-wire group versus 7.25 ± 5.1 degrees in the lateral-wire group (p = 0.9567).

AHL was not intersecting the capitellum when comparing intra-operative and postoperative follow-up X-rays in six (15.2%) and nine (27.6%) in the crossed and lateral wire groups, respectively.

In our study, overall higher (19, 46.3% vs. 5, 14.7%) complications were noted in the crossed-wire group in comparison to the lateral wire, and it was statistically significant (p = 0.0052).

Overall, iatrogenic nerve injury was noted in 10 (13.3%). In the crossed-wire group, three (7.3%) children suffered iatrogenic ulnar nerve injury, but none in the lateral group. Anterior interosseous nerve (AIN) injury was the most common nerve injury, with three cases observed with crossed-wire fixation. Different types of nerve injury for both groups are shown in more detail below in Table 4. The crossed-wire group had significantly higher iatrogenic nerve injury than the lateral wire group, 9 vs. 1 (22% vs. 2.9%, p = 0.0185). However, most were resolved fully in 12 weeks, except for one case. This case was referred to a specialist center for further management.

Though statistically insignificant, higher complications, such as pin site infection, hyperextension, loss of functional ROM, and cubitus varus deformity, were noted in the crossed-wire group. This, in part, could be due to a significantly higher number of Gartland type III fractures and requirements for open reduction in the crossed-wire group.

A superficial pin site infection treated with antibiotics was only seen in the crossed-wire group 3 (7.3%). No deep-seated infections were observed, and no revision surgery was required for infection. Similarly, one case (2.4%) of hyperextension of the elbow and one case (2.4%) of cubitus varus deformity were only noted in the crossed-wire group, but none in the lateral group. One patient (2.9%) had revision fixation within three months in the lateral wire group but none in the crossed-wire group. Loss of functional ROM and significant stiffness of the elbow were seen in five cases (12.2%) and three cases (8.8%) in the crossed wire and lateral wire groups, respectively. This data is presented below in Table 5.

**Table 5:** Complications associated with crossed and lateral K-wire fixation A p-value is considered significant (p < 0.05); significant p-values are denoted with *. Complication variables are presented as N (%).

Complications	Crossed wire (n = 41) N (%)	Lateral wire (n = 34) N (%)	p-value
Overall complications	19 (46.3%)	5 (14.7%)	0.0052*
Iatrogenic nerve injury	9 (22%)	1 (2.9%)	0.0185*
Ulnar nerve (UL)	2 (4.9%)	0	-
Radial nerve (RN)	1 (2.4%)	0	-
Medial nerve (MN)	2 (4.9%)	0	-
Anterior interosseous nerve (AIN)	3 (7.3%)	0	-
Combination and types	1 MN + UL (2.4%)	1 AIN + MN (2.9%)	-
Revision fixation or reoperation within three months	0	1 (2.9%)	-
Pin site infection	3 (7.3%)	0	-
Significant stiffness elbow (loss of functional ROM)	5 (12.2%)	3 (8.8%)	-
Hyperextension	1 (2.43%)	0	-
Cubitus varus	1 (2.4%)	0	-

## Discussion

In our series, 17 (22.7%) cases needed open reduction due to failure of closed reduction, and the majority (14, 82.4%) had crossed-wire fixation. The rate of open reduction varies from 3% to 46% in the literature [6,7]. Traditionally, it has been reserved for cases with primary vascular injury, open fractures, and failure of closed reduction. Historically, there was a high incidence of complications, e.g., stiffness or myositis ossificans, associated with an open approach. However, in more recent studies, there were no differences in loss of motion, infection, malunion, or subsequent surgery compared with a closed reduction [8].

In our cohort, overall rates of open fracture were one (1.3%), preoperative nerve injury were three (4%), and concurrent fractures were two (2.7%), which are consistent with those reported in the literature [9-11].

Percutaneous fixation using K-wires is widely accepted as a treatment modality, but ideal pin placement is still being debated. Our study found no statistical difference in the radiographic loss of reduction between the crossed and lateral wire fixation groups (p = 0.926).

The results of the studies in the literature are controversial with regard to bio-mechanical stability. Whilst some studies suggest that crossed wiring increases stability, other studies have not found any differences [12,13]. There is no statistically significant difference between using three laterally placed wires and crossed wires, despite the latter being technically bio-mechanically superior. When more stability is needed, three lateral wires are often recommended to reduce the risk of iatrogenic ulnar nerve injury [14,15].

**Figure 6:**
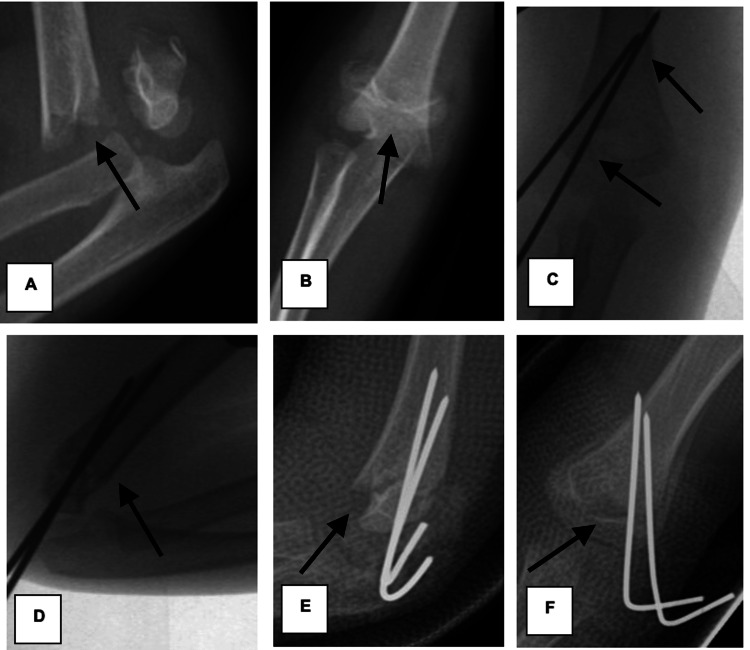
Inadequate fixation using lateral wires leading to postoperative loss of reduction from one of our cases (A, B) Preoperative AP and lateral radiographs of Gartland type III supracondylar fracture. (C, D) Intraoperative AP and lateral radiograph with 2 lateral wires showing adequate reduction but inadequate fixation (not bi-columnar, not divergent, separation of wires <1/3 circumference of the humerus at the fracture site). (E, F) Three weeks postoperative AP and lateral radiographs demonstrating loss of reduction. AP: anteroposterior

In 2007, Zenios et al. explained the technical difficulty surrounding the adequate placement of lateral wires, with a proposition of a protocol of standardized fixation using two lateral wires [16]. If still unstable, a third lateral wire was placed. If the fracture was still unstable, a medial wire was placed. Larson et al. demonstrated that three divergent lateral wires are at least as stable as conventional crossed wires [17]. According to Skaggs et al., even the most unstable fractures can be treated successfully with just lateral pinning [4]. In our cohort, technical error of wire fixation was noted in 28 (82.4%) and 12 (29.3%) of the lateral and crossed-wire fixation groups, respectively. This was statistically significant (p = 0.00001). The most common error in the lateral group was an inadequate separation of wires (<1/3 of humerus width at the fracture site) in 24 (85.7%) of cases, followed by non-bi-columnar wires in 22 (78.6%). In the crossed-wire group, the most common error was the lack of bi-cortical wire purchase in nine (75%) cases. We noted a 27% higher loss of reduction associated with technical errors of lateral wire fixation, but statistically, it was insignificant (p = 0.254). An example of technical errors with lateral wire fixation leading to loss of reduction postoperative is depicted below in Figure 6 from one of our cases. Higher loss reduction of fracture with faulty lateral wire placement was reported in the literature [4,5].

Sankar et al. explained and described pinning errors as types A, B, and C. Type A errors were those that failed to engage both fragments by two pins or more. Failure to achieve bi-cortical fixation with two pins or more was referred to as a type B error. Inadequate pin separation to control rotation was referred to as a type C error [18].

In our series, higher complications were observed in the crossed-wire group compared to the lateral wire, 19 versus 5 (46.3% vs 14.7%, p = 0.0052). Nine (47%) of these complications in the crossed-wire group were due to iatrogenic nerve injury.

In our cohort, overall nerve injury (preoperative and iatrogenic) was seen in 13 (17.3%), with the AIN most commonly affected, followed by the median nerve. The incidence of nerve injuries in supracondylar fractures varies from 6.5% to 19%, and AIN is most commonly involved in extension-type fractures [19,20]. Combined nerve injury may be observed in up to 21% of cases with nerve lesions [21].

In our series, preoperative nerve injury was documented in only three (4%) cases. The majority of injuries come from fracture displacement, which can stretch, entrap, or disrupt nerves. In our series, documented preoperative nerve injury was less than noted in the reported literature and could be due to inadequate preoperative assessment, documentation, or both. In our series, 10 (13.3%) had iatrogenic nerve injuries. The most common causes of iatrogenic nerve injury include excessive manipulation, immobilization in hyperflexion, or the insertion of wires [20,21].

The majority of the iatrogenic nerve injuries were noted in our crossed-wire group (9, 22%), whereas there was only one case (2.9%) in the lateral wire group. In our series, iatrogenic nerve injury is higher than reported in the literature. This may be due to inadequate assessment and documentation of individual nerve function during the initial presentation in the emergency department, indicating that a proportion of these iatrogenic injuries may, in fact, have been preoperative. Recently, the British Orthopaedic Association Standards for Trauma (BOAST) mentioned, in the updated version of October 2020, the importance of documentation of individual nerve assessment at presentation and just before surgery. Moreover, higher numbers of type III fractures were noted in the crossed-wire group, with a higher requirement for open reduction. This may be connected to a higher incidence of iatrogenic nerve damage in the crossed-wire group. In the crossed-wire group, we found three cases (7.3%) with ulnar nerve damage, but none in the group with lateral wire fixation. When crossing pins are used instead of lateral entry points alone, numerous studies have demonstrated an increased incidence of ulnar nerve injury, with no differences in other parameters. Crossed pins have a higher relative risk (RR) of damage to the ulnar nerve than lateral-entry pins by a factor of up to 4.3 [22].

In our opinion, the best way to avoid iatrogenic ulnar nerve injury is to avoid crossed wiring. However, crossed wires can be considered when an open reduction of a fracture is performed through a medial approach in the presence of significant medial comminution, medial high oblique fractures, high transverse fractures, or persistent instability after three lateral pins.

In our series, most cases of nerve injuries are resolved fully by 12 weeks. In their study, Valencia et al. also noted that in the long-term follow-up, 100% of radial nerve injuries, 87.5% of median nerve injuries, and 25% of ulnar nerve injuries totally recovered with conservative management [21]. The median nerve recovered on average in 2.5 months, the ulnar nerve in five months, and the radial nerve in three months on average [21].

A change in the vascular status occurs in 10% to 20% of displaced supracondylar fractures [23]. About 7% to 12% of all fractures and up to 19% of displaced fractures are found to have no radial pulse prior to reduction. We observed two (2.7%) individuals who presented with abnormalities in vascular status, which is fewer than reported in the literature. The neurovascular structures on the proximal segment can be stretched, entrapped, or disrupted, leading to lesions of the brachial artery. Secondary causes include reduction maneuvers and immobilizing the elbow in the hyper-flexed position [20,23].

In our series, one (2.9%) of the patients, only in the lateral wire group, required revision surgery as a result of insufficient fixation and loss of reduction.

There were three (7.3%) cases who suffered superficial pin site infection in the crossed-wire group, which resolved completely with oral antibiotics. There were no superficial pin site infections in the lateral wire group. The incidence of pin-track infections in the literature varies from 1% to 25% [24]. Most infections are superficial and improve with oral antibiotics and pin removal [4,25]. One patient (2.4%) had a hyperextension-type deformity in our crossed-wire group. It is commonly related to the healing of fractures in extension. An extension malunion results in a loss of flexion and an increase in extension, causing notable cosmetic problems. Therefore, careful reduction is crucial to avoid extension malunion. In our study, one (2.4%) patient had a documented cubitus varus deformity in the crossed-wire group. Cubitus varus is believed to be caused by angular and rotational deformities. According to research by Labelle et al., children who underwent surgical correction of their cubitus varus exhibited no functional differences, indicating that the procedure was performed to enhance cosmesis alone [26].

In our series, significant stiffness in the elbow, measured by a loss of functional range of motion (ROM), was noted in eight (10.7%) cases. It was slightly higher in the crossed-wire group, and this was statistically insignificant. About 30-130 degrees ROM is considered the functional ROM in the elbow. Most patients with supracondylar fractures experience stiffness, which is quite apparent once the plaster cast is removed. In our experience, most children regained their full ROM with time. However, in our study, a physiotherapy referral was done after the removal of the K-wires in 16 (39%) and 15 (44.1%) cases in the crossed-wire and lateral-wire groups, respectively (p = 0.814). According to research by Ducic et al., children who received physiotherapy showed faster improvement in ROM in the initial few months but no change after 12 months. Therefore, routine physiotherapy referral is not advisable in every case of supracondylar fractures, and we do not routinely refer children to physiotherapy following supracondylar fractures [27].

Limitations

Our study includes several limitations. The retrospective design is susceptible to selection bias. It is a retrospective study where a number of different surgeons carried out the procedures. The lack of a standardized protocol resulted in the size of pins and pin configuration being dependent on the surgeon’s choice. Also, we have included all displaced supracondylar fractures, both Gartland type II and type III treated with pinning, rather than focusing on only one type of fracture; this may affect our study's radiographic outcomes or complications. We had higher numbers of type III fractures and a higher rate of open reduction in crossed-wire groups than in lateral-wire groups. Furthermore, with a longer follow-up period, further complications could have been observed. However, we feel the salient complications in this cohort are immediate or early and comprehensively covered in this study.

## Conclusions

We observed higher complications in the crossed-wire compared to the lateral wire fixation group, primarily due to a higher rate of iatrogenic nerve injury. We did not find any statistically significant difference in radiographic loss of reduction and various complications between the two groups, including infection, stiffness, cubitus varus, hyperextension, and reoperation or revision fixation within three months. Incidences of vascular injury or compartment syndrome requiring surgery are rare complications following displaced supracondylar fractures.

The correct lateral wiring technique is slightly more challenging than the crossed wiring technique. We observed significantly higher technical errors in lateral wire fixation and higher loss of reduction with such technical errors. To evaluate the associations between radiographic loss of reduction and clinical outcomes in both groups, further randomized controlled studies should be conducted with a sufficient number of patients. To avoid iatrogenic nerve injury and other complications, we support lateral wiring with the correct technique in treating displaced supracondylar humeral fractures in children. However, a larger sample size is necessary, along with additional research entailing meticulous data collection and research methodology to conclude anything definitively.
